# Phytoplankton blooms in Lake Winnipeg linked to selective water-gatekeeper connectivity

**DOI:** 10.1038/s41598-019-44717-y

**Published:** 2019-06-10

**Authors:** Genevieve Ali, Carolyn English

**Affiliations:** 1 School of Environmental Sciences, University of Guelph, Ontario, Canada; 2 Department of Geological Sciences, University of Manitoba, Manitoba, Canada; 3 Watershed Systems Research Program, University of Manitoba, Manitoba, Canada

**Keywords:** Environmental impact, Hydrology

## Abstract

Lake Winnipeg was coined “Canada’s sickest lake” and “the most threatened lake in the World” due to its recurrent algal blooms caused by nutrient-rich water inputs. While conceptual frameworks link bloom occurrence to hydrologic connectivity, data-based validation is lacking. We analyzed 355 multi-year satellite-derived images to quantify phytoplankton biomass in Lake Winnipeg and the timing of runoff activation and hydrologic connectivity in the Lake Winnipeg Watershed. Our analyses reveal that the majority of watershed runoff-producing areas exhibit a strong coupling between runoff activation and hydrologic connectivity: they are proximal to rivers and become hydrologically connected to them multiple times a year. Conversely, a smaller number of runoff-producing areas are located further upslope and connect to rivers much less frequently. The latter act as water gatekeepers by selectively enabling the downstream transfer of runoff from headwater regions. Major blooms in Lake Winnipeg only occur when 50% of the water gatekeepers enable headwater-downstream connectivity during 31.5% (or more) of the spring-fall period. We conclude that an explicit assessment of the timing of runoff activation and hydrologic connectivity serves as a predictor of bloom occurrence and provides new information about the influence of a small number of locations on Lake Winnipeg.

## Introduction

**Figure 1:**
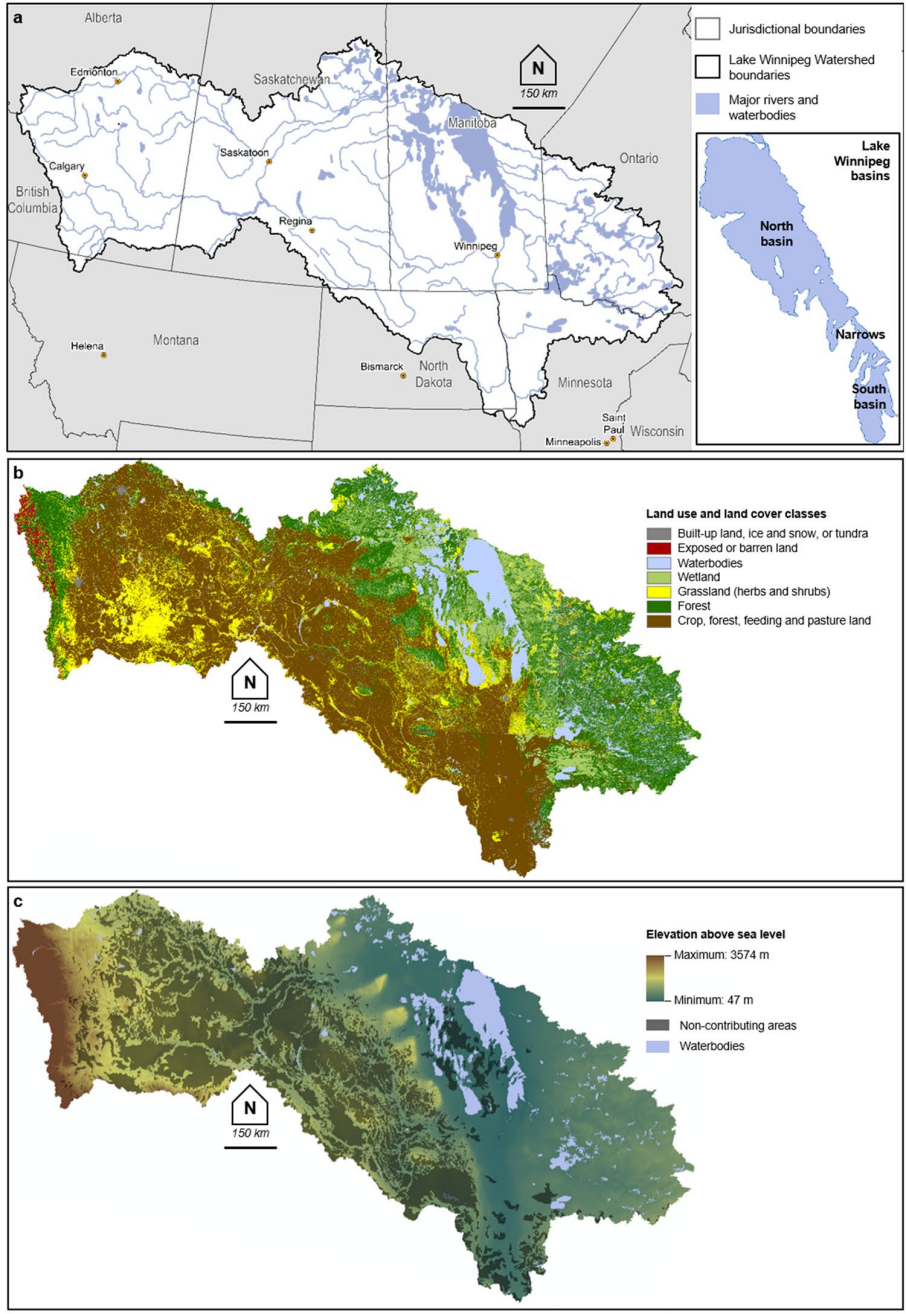
Lake Winnipeg Watershed characteristics. (**a**) Location of Lake Winnipeg, its watershed and its main rivers in North America. The north and south basins of the lake are identified. (**b**) Land use and land cover types across the watershed. (**c**) Elevation and (historically labelled) non-contributing areas across the watershed. Physiographic data (i.e., rivers and waterbodies, digital elevation data) and land use and land cover data were obtained from public repositories administered by the Government of Canada (see Methods) and mapped using the ArcGIS software (version 10.4.1, www.esri.com).

Excess amounts of nutrients lead to eutrophication, which is the leading cause of impairment of inland waters, estuaries and coastal waters around the World^1^. One well-known example of a eutrophic lake is Lake Winnipeg^2–4^, which is the 10^th^ largest freshwater lake, by surface area, globally^1,5,6^. Notable characteristics of Lake Winnipeg include an overall shallow depth and the presence of two basins (i.e., north and south) connected by a narrow channel^5^ (Fig. 1a). Heterocytous cyanobacteria, also known as blue-green algal blooms, have increased not only in occurrence but also in extent in the lake since the 1990s^2,7^. The increase has been attributed to the combined effect of cultural eutrophication that is a consequence of over 50 years of intense agricultural and other human activities in the region^2,6,8,9^, as well as climate-related eutrophication that is caused by higher-frequency floods enhancing the runoff-driven mobilization of phosphorus and nitrogen from the Lake Winnipeg Watershed^2,6^. As phosphorus is the main nutrient of concern, a 50% phosphorus loading reduction goal has been set in an attempt to restore Lake Winnipeg back to a “less eutrophic” state^2^.

While several strategies are adopted around the World to mitigate eutrophication, they can be difficult to implement in lakes associated with very large and heterogeneous watersheds^8^. Enclosed within the Lake Winnipeg Watershed is nearly 1,000,000 km^2^ of land spanning Canada and the United States (Fig. 1a), with sparse human population but intensive human activities, including agriculture^5^ (Fig. 1b). Water quality issues in the rivers and lakes of the region are attributable to a combination of factors. Although wastewater and sewage lagoons are non-negligible nutrient sources, it is regional soil characteristics that make phosphorus loss to surface waters widespread and, thus, a major land and water management issue^10^. Saturated and flooded agricultural soils increase the mobilization of phosphorus from land to streams^6,11^, while freeze-thaw cycles further enhance phosphorus release from grassland vegetation and crop residues^12^. Intensively managed watersheds such as the Lake Winnipeg Watershed have large soil nutrient stores^13,14^ and hence, their ability to export nutrients to downstream waters is not limited by nutrient availability but rather by hydrologic transport mechanisms^15^.

Previous studies on soil phosphorus, river flow, river phosphorus concentrations and lake chlorophyll-a concentrations have established strong correlations between watershed runoff dynamics, phosphorus desorption from soils, nutrient loading to rivers and lakes, and significant algal blooms in Lake Winnipeg^6^. Identifying runoff sources and their timing of activation in this region is, however, a challenge due to seasonal and sub-seasonal changes in runoff generation processes. While 60% to 80% of the annual runoff occurs during snowmelt, the alternation of cold, dry and flood conditions allows for a range of runoff generation mechanisms, from infiltration-excess overland flow to saturation-excess overland flow and shallow subsurface flow^14^. Low-relief landscapes across the majority of the Lake Winnipeg Watershed (Fig. 1c), combined with depressional wetlands and hummocky terrain in the western and central portions of the watershed, respectively, signify that partial area dynamics and threshold-driven fill-spill processes dominate while traditional variable source area concepts are seldom applicable. Instead of variable source areas, large parts of the watershed were historically labelled as non-contributing areas (Fig. 1c): akin to closed basins, they are thought not to transmit surface runoff to proximal streams under normal conditions (i.e., events with a 2-year return period)^16^.

One concept allowing the comprehensive assessment of hydrologic transport mechanisms, in terms of local runoff production but also runoff and contaminant transmission from land to rivers, is that of hydrologic connectivity. Hydrologic connectivity describes the degree to which disparate landscape locations exchange water (and its associated chemical constituents), and it is appropriate to refer to regardless of the flowpaths under consideration, namely surface (above ground), shallow subsurface (just below the ground surface), and/or deep subsurface (through deep underground formations and bedrock) flowpaths^17^. While the scientific community is still searching for the best way to assess multi-flowpath hydrologic connectivity^18,19^, spatial metrics can be used to quantify the timing of surface or shallow subsurface connectivity from soil moisture maps^20,21^. Computing these metrics involves classifying landscape areas as either hydrologically active, i.e., producing surface or shallow subsurface runoff, or inactive, and then assessing the spatial adjacency of hydrologically active areas to the river network. No previous study has examined temporal changes in the spatial extent of hydrologically active areas across large lake watersheds, nor the frequency with which those areas connect to the river network and the relationship those dynamics have with algal or phytoplankton bloom development in receiving lakes. Using the iconic Lake Winnipeg as an example of a eutrophic lake, here we directly explore the links between the timing of watershed hydrologic connectivity and lake phytoplankton blooms by combining 165 satellite-derived images of near-surface soil moisture with 190 satellite-derived images of lake water chlorophyll-a concentrations (see Methods). As the images span eight different years and capture quasi-weekly conditions during the open-water, ice-free period, we use them to link the occurrence and duration of hydrologic connectivity to phytoplankton bloom development in Lake Winnipeg on an annual basis.

## Results – Phytoplankton Blooms

**Figure 2:**
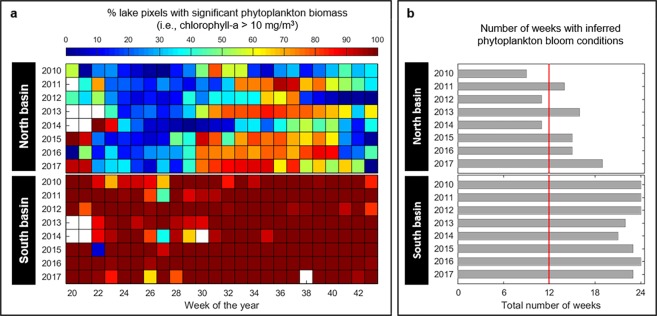
Phytoplankton blooms in Lake Winnipeg. (**a**) Weekly assessment of the percent lake pixels covered by significant phytoplankton biomass for the north and south basins of Lake Winnipeg. (**b**) Yearly number of weeks with phytoplankton bloom conditions. The red line signals the half-way mark (12 weeks) of the open water, ice-free period. The plots were created using MATLAB (version R2016a, www.mathworks.com).

Data show that between May 2010 and October 2017, the range of chlorophyll-a concentrations observed in the north and south basins of Lake Winnipeg was similar, namely 1–99 mg/m^3^ and 0–99 mg/m^3^, respectively. Weekly mean chlorophyll-a concentrations were, however, different, averaging 13 mg/m^3^ for the north basin and 32 mg/m^3^ for the south basin. Those variations are important since the presence of significant phytoplankton biomass in Lake Winnipeg has been inferred from satellite imagery when chlorophyll-a concentrations were equal to 10 mg/m^3^ or more^22–24^. On average, 42.9% of the surface area of the north basin was covered by significant phytoplankton biomass (Fig. 2a), as opposed to 95.8% of the south basin (see Methods). Seasonal differences were present for the north basin: the spatial extent of significant phytoplankton biomass was, typically, at a minimum in late spring and early summer (weeks 20–28) and at a maximum in late summer (weeks 29–35). With the exception of the years 2010, 2012 and 2014 for the north basin, Lake Winnipeg experienced bloom conditions for more than half of the ice-free, open water period (i.e., more than 12 out of 24 weeks) (Fig. 2b). Based on the temporally-varying conditions in the north basin, the years 2010, 2012 and 2014 were labelled as moderate-bloom years in Lake Winnipeg, while the years 2011, 2013, 2015, 2016 and 2017 were labelled as major-bloom years.

### Results – Runoff activation and hydrologic connectivity

**Figure 3:**
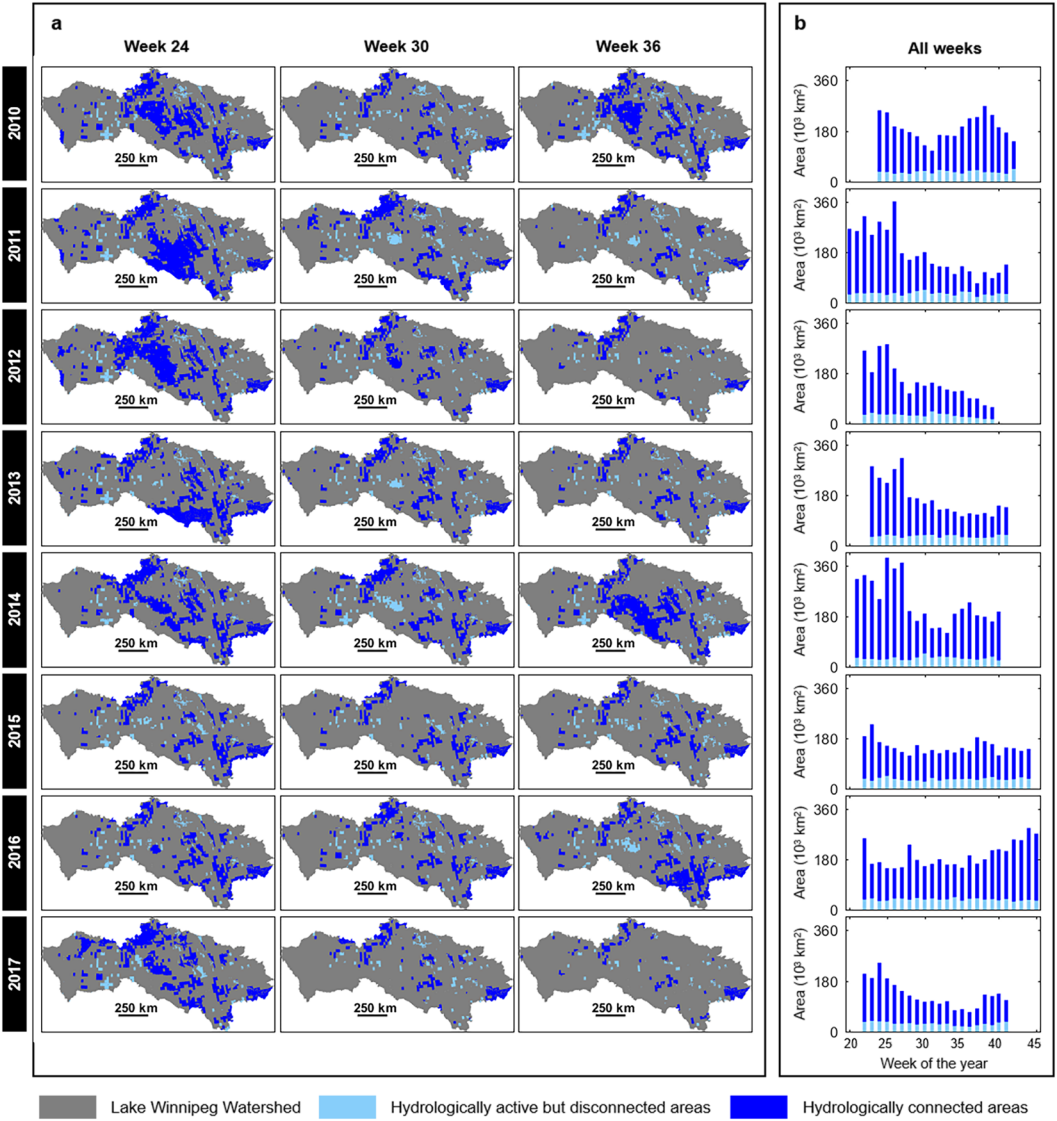
Temporal variability of near-surface runoff activation and hydrologic connectivity in the Lake Winnipeg Watershed. **(a**) Maps of hydrologically active but disconnected, and hydrologically connected areas across the whole Lake Winnipeg Watershed for selected weeks during the 2010–2017 period. For additional information, the 165 maps of hydrologically connected areas used in this paper can be visualized as a slideshow made available online (see Supplementary Video). (**b**) Weekly total extent of hydrologically active but disconnected, and hydrologically connected areas for the 2010–2017 period. The absence of bars signals missing data. The plots were created using MATLAB (version R2016a, www.mathworks.com).

**Figure 4:**
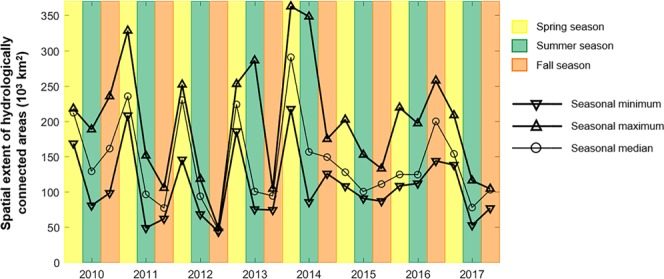
Seasonality effects on connectivity. Changes in the minimum, maximum and median spatial extent of hydrologically connected areas in the Lake Winnipeg Watershed across the spring, summer and fall seasons during the 2010–2017 period. The plots were created using MATLAB (version R2016a, www.mathworks.com).

**Figure 5:**
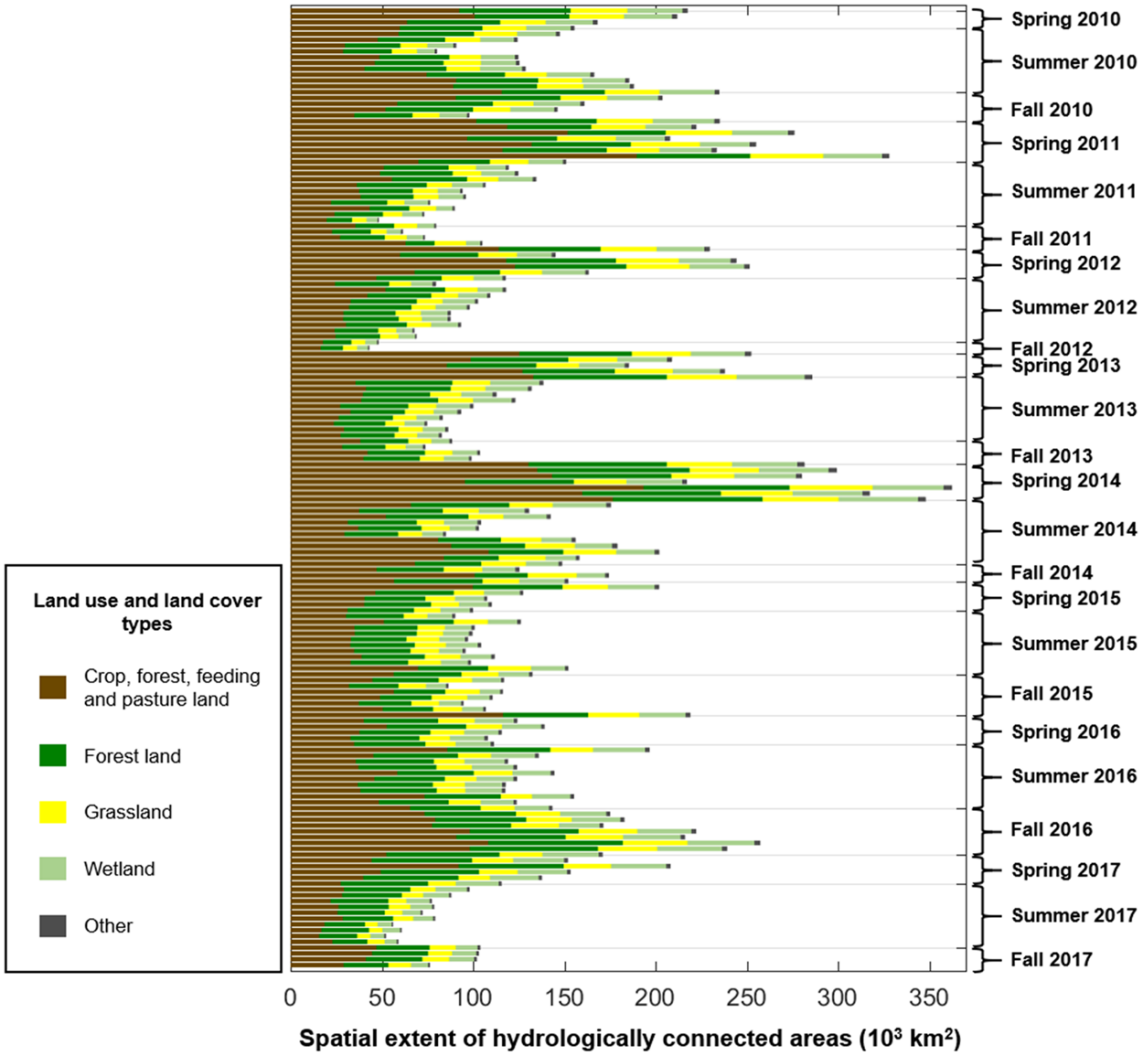
Land use and land cover controls on connectivity. Distribution of hydrologically connected areas across the main land use and land cover types present in the Lake Winnipeg Watershed. Each horizontal bar is associated with a different, weekly map during the 2010–2017 period. The plots were created using MATLAB (version R2016a, www.mathworks.com).

Between May 2010 and October 2017, there was also a lot of variability in the areas of the Lake Winnipeg Watershed classified as (i) hydrologically inactive; (ii) hydrologically active (i.e., runoff-producing but geographically isolated from the river network); and (iii) hydrologically connected (Fig. 3). Depending on the date considered, the extent of hydrologically inactive areas ranged between 65% and 95% of the overall Lake Winnipeg Watershed area. Those estimates, while large, are not surprising given the semi-arid to sub-humid climate prevailing across the watershed and the presence of soils which, for the most part, have a large water infiltration and storage capacity^14^. The western and central portions of the Lake Winnipeg Watershed are also known for their numerous topographic depressions: they host pothole wetlands and small lakes which spill over their rims to trigger surface runoff only under very wet and flooding conditions^25,26^. Under non-flooding conditions, these topographic depressions act as long-term water stores in a landscape that does not otherwise produce surface or shallow subsurface runoff, causing widespread hydrologic inactivity and thus disconnectivity^16,27^. As for hydrologically connected areas, which not only produce surface or shallow subsurface runoff but are also spatially contiguous to major rivers (see Methods), they were typically the most extensive in mid- to late May (week 24) and the least extensive in fall (e.g., week 36; see Figs 3a and 4 and Supplementary Video). The spatial extent of hydrologically connected areas ranged between 43,244 km^2^ (~4% of the Lake Winnipeg Watershed) and 362,555 km^2^ (~36% of the Lake Winnipeg Watershed) (Fig. 3b), with the highest values recorded in spring 2011 and early summer 2014 (Fig. 3b). Those highest values coincide with flood conditions in Saskatchewan and Manitoba^28,29^ that were triggered by heavy snowmelt on saturated soils in 2011, and by unusual frontal systems causing long-duration rainfall on saturated soils and on topographic depressions already (or close to being) filled to capacity in 2014. The spatial extent of hydrologically active but disconnected areas was small, compared to that of hydrologically connected areas, and relatively stable in time (Fig. 3b). Across all seasons during the 2010–2017 period, the vast majority of hydrologically connected areas were located in agricultural land and grassland (Figs 3a and 5), especially in the western and central portions of the Lake Winnipeg Watershed (Fig. 1c). Hydrologically connected areas in the eastern portion of the Lake Winnipeg Watershed were rather located in forest land (Figs 1c and 5).

**Figure 6:**
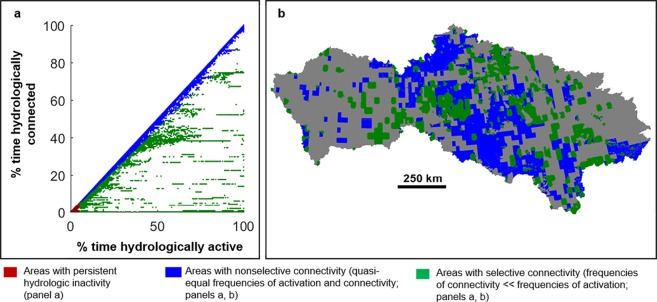
Typology of connectivity establishment in the Lake Winnipeg Watershed. (**a**) Classification of watershed areas as persistently inactive (% time with hydrologic activity <5%), with nonselective connectivity (i.e., % time hydrologically active ≈ % time hydrologically connected), and with selective connectivity (% time hydrologically connected ≪% time hydrologically active). (**b**) Identification of non-selectively and selectively connected areas within the Lake Winnipeg Watershed. For better clarity, persistently inactive watershed areas are not shown in red on the map. The plots were created using MATLAB (version R2016a, www.mathworks.com).

Several watershed areas were deemed hydrologically inactive for more than 95% of our study period (Fig. 6a), signalling that they were either rarely producing any runoff or that they were producing runoff via hydrological processes, such as deep subsurface flow, not captured by near-surface soil moisture data. In contrast, other areas regularly changed status between inactive, active and connected. Two distinct types of behaviour were observed based on the coupling (or decoupling) of runoff activation (i.e., hydrologic activity) and hydrologic connectivity. On one hand, for a large majority of near-river areas, there was a very strong agreement, shown by high correlation and a slope of 0.9 to 1, between the duration of hydrologic activity and that of hydrologic connectivity (Fig. 6a). Due to their proximity to the river network (Figs 1a and 6b), for those areas hydrologic activity automatically led to hydrologic connectivity. We refer to that strong coupling between runoff activation and hydrologic connectivity as nonselective connectivity. On the other hand, for a smaller number of areas, there was no correlation between the duration of hydrologic activity and that of hydrologic connectivity (Fig. 6a), which suggests that connectivity was selectively established at specific times. In those cases, hydrologic activity was a necessary but not a sufficient condition for connectivity to occur. The main physiographic differences between non-selectively and selectively connected areas were not in terms of land use and land cover but rather in terms of geomorphic position. Indeed, both types of connected areas were mostly associated with agricultural land (in a proportion of 45–51%), followed by forest land (24%), grassland (12–15%) and wetland (11–14%). A large proportion (i.e., 38–41%) of connected areas were located in the closed basins highlighted in Fig. 1c, thus highlighting discrepancies between the temporally static classification of non-contributing areas that was established historically^16^ and the temporally dynamic, soil moisture-based assessment of connectivity done in the current study. Selectively connected areas were often located at the immediate periphery of non-selectively connected areas and in more distant floodplain and headwater areas (Figs 1a and 6b).

### Results – Relationship between phytoplankton blooms and hydrological dynamics

**Figure 7:**
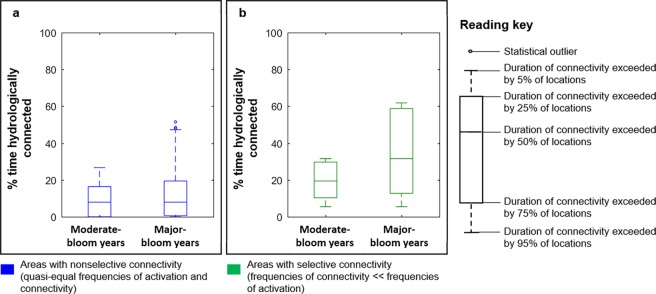
Linkage between connectivity duration and lake phytoplankton blooms. (**a**) Differences in connectivity duration between moderate-bloom years and major-bloom years for non-selectively connected areas only. (**b**) Same analysis but for selectively connected areas only. The plots were created using MATLAB (version R2016a, www.mathworks.com).

A range of Kruskal-Wallis tests were performed to evaluate differences in runoff activation and connectivity between moderate-bloom years and major-bloom years. In all cases, the magnitude of the Kruskal-Wallis test statistic, H, was very large given the very large datasets analyzed. The tests yielding the largest H-statistic values and the smallest p-values were interpreted as particularly significant. Across all watershed locations, the median percent time with runoff activation was only 0.6% higher in major-bloom years, although that difference was statistically significant (H-statistic = 4692, df = 1, p-value = 0). Similarly, the difference in overall median connectivity duration between moderate-bloom years and major-bloom years was also less than 1%, albeit statistically significant (H-statistic = 3694.19, df = 1, p-value < 0.0001). Distinguishing between non-selectively connected areas and selectively connected areas led to strikingly different conclusions regarding the impact of watershed hydrology on bloom development in Lake Winnipeg. When considering non-selectively connected areas only, there was an almost undiscernible, albeit statistically significant, difference (Kruskal-Wallis test; H-statistic = 1246.43, df = 1, p-value < 0.0001) in the duration of connectivity between moderate-bloom years and major-bloom years (Fig. 7a). A large, statistically significant difference was, however, present when considering selectively connected areas (Kruskal-Wallis test; H-statistic = 33755.85, df = 1, p-value = 0): major blooms occurred when 50% of the selectively connected areas were hydrologically connected for more than 31.5% of the ice-free, open-water period, as opposed to only 19.4% of the ice-free, open-water period during moderate-bloom years (Fig. 7b). Further, in major-bloom years, 25% of the selectively connected areas were hydrologically connected for 58.8% of the ice-free, open-water period, as opposed to 29.7% of the ice-free, open-water period during moderate-bloom years (Fig. 7b).

## Discussion and Conclusions

Our assessment of runoff-producing areas was based on near-surface (<5 cm depth) soil moisture maps and as such, it infers runoff activation and hydrologic connectivity created by saturation-excess overland flow or shallow, saturated subsurface flow. This assessment is, therefore, conservative, as it may not have captured instances of infiltration-excess overland flow that can also occur, either as sheet flow of meltwater over frozen ground during the first days of the annual spring freshet, or as rapid surface runoff above unsaturated soils following short-duration, high-intensity convective rainstorms in summer^14^. As for our assessment of bloom conditions, it considered all chlorophyll-bearing biomass (including diatoms) and not solely cyanobacteria, a drawback frequently encountered when using satellite-derived images to characterize lake eutrophication^30^. Nonetheless, our study shows important temporal variability in watershed as well as lake dynamics. Bloom extent varied from week to week and year to year (Fig. 2), which has been previously observed in Lake Winnipeg; it was attributed to a range of internal factors such as lake circulation patterns and water temperature, as well as external factors such as phosphorus loadings from the watershed^6,9^. The total spatial extent of hydrologically connected areas also varied (Fig. 3), which was to be expected in the Lake Winnipeg Watershed where both the seasonality and the inter-annual variability of wet and dry spells affect runoff generation and runoff transmission losses. Flood periods were identified as the ones with the highest degree of watershed hydrologic connectivity.

In legal debates surrounding land and water management, many have argued that the implementation of connectivity-based policy is challenging due to a lack of convincing, field-based evidence^19,31,32^ showing the link between watershed-wide runoff connectivity and the health of downstream aquatic systems such as lakes. Furthermore, regulatory discussions of connectivity still mostly focus on distance criteria or river adjacency criteria^33^, with the assumption that only near-river areas exert a significant and quantifiable influence on downstream systems. Our study not only challenges those preconceptions but also provides the first quantitative estimates of connectivity duration leading to major phytoplankton blooms in Lake Winnipeg (Fig. 7). Indeed, while studies linking river inflows and nutrient loads to lake algal blooms are common^34–36^, spatially detailed investigations aiming at identifying the specific watershed areas that contribute most to river flows, and hence to nutrient loads, are rare to nonexistent. It is worth noting that hydrologic connectivity, as a concept, can be quantified through a range of properties, namely its occurrence (which is a binary measure), its spatial extent (which is critically important for targeted land management), its frequency or duration (which can be linked to the frequency or duration of other hydrometeorological processes), and its magnitude (which illustrates the volume of water delivered from a source to a receptor point)^19^. The lack of comprehensive, publicly available datasets did not allow for a comparison of watershed areas in terms of connectivity magnitude (i.e., runoff volumes and associated nutrient loads) and its impact on bloom occurrence. Similar to other connectivity studies, however, our focus was not on the mechanisms responsible for runoff activation and hydrologic connectivity but rather on if, when and for how long connectivity was detected^20,21,37^.When it comes to the duration of runoff activation and connectivity, our analysis showed that non-selectively connected areas did not exhibit very different behaviors between moderate-bloom and major-bloom years, as opposed to selectively connected areas: that observation gives credence to conceptual frameworks which have argued that intermittent or ephemeral connections can have significant environmental effects on downstream water quality^38^. The fact that the majority of selectively connected areas are not adjacent to major rivers also gives credence to previous studies which have highlighted the key role of hillslopes and non-floodplain upland areas, including wetlands, seasonally flowing streams and other types of “vulnerable waters”, in driving connectivity^38–40^. We hypothesize that selectively connected areas act as water gatekeepers^26^. In a broad sense in network theory, gatekeepers are defined as having a position in a network that is critical for the overall connectivity of the network. In our study, selectively connected areas appear to behave as such: while they allow the transmission of runoff from headwaters to major rivers when they are active, they prevent that transmission from occurring either when they are inactive, or when they are active but hydrologically disconnected from the river network.

It should be noted that the water gatekeepers identified in this study span less than 10% of the whole Lake Winnipeg Watershed area. This statistic could be significant if confirmed, as it would imply that a small number of locations with selective connectivity establishment can play an important role in nutrient mobilization from a watershed and, as a result, the severity of phytoplankton blooms in a lake at the downstream end of said watershed. That important role may be through the runoff-driven mobilization of nutrients from the gatekeeper locations themselves, or through the export of runoff water from the gatekeeper locations to allow the enhanced mobilization of nutrients further downstream, *en route* to the lake. The extent to which these conclusions can be generalized remains, however, to be determined. For Lake Winnipeg and its watershed, there is a possibility that slightly different conclusions could be reached if different data relying on different satellite-mounted imaging sensors with different spatial resolutions are used. More importantly, no study has examined whether the hydrological status of water gatekeepers can be correlated to major blooms in other great lakes such as Lakes Erie, Ontario or Okeechobee. Meanwhile, our analysis bears important implications for land and water management in the Lake Winnipeg Watershed. Given the large land mass associated with the lake watershed, achieving a 50% phosphorus loading reduction goal will be difficult unless targeted action is directed at regions deemed most critical for the establishment of hydrologic connectivity. The selectively connected areas, or water gatekeepers, identified in our analysis are such critical areas: they offer tangible opportunities to actively manage surface and near-surface water, through man-made water stores, for instance, so as to purposefully establish hydrologic disconnectivity and thus prevent runoff and associated contaminants from reaching the Lake.

## Methods

Our study focuses on Lake Winnipeg and its watershed. To characterize phytoplankton blooms in the lake, MODerate resolution Imaging Spectroradiometer (MODIS) data, specifically MODIS/Terra Level 3 chlorophyll-a data, were used. Those data, available globally, take the form of composite gridded images with approximate 4-km spatial resolution. The chosen composite images summarize data averaged over an 8-day period (and hence multiple satellite passes) with minimum cloud contamination. The rationale for using MODIS/Terra Level 3 images was twofold: (i) the open access nature of the data, and (ii) the fact that the MODIS/Terra sensor was launched close to two decades ago and is still operating. MODIS/Terra chlorophyll-a images were downloaded from the NASA Ocean Color website (https://oceandata.sci.gsfc.nasa.gov/; maintained and provided for use by NASA Goddard Space Flight Center, Ocean Biology Processing Group; last accessed: November 2017). MODIS/Terra chlorophyll-a images were processed in MATLAB solely for the geographic region corresponding to Lake Winnipeg (latitude: 50–54.0°N; longitude: 95–100°W). Images produced for the period May 2010-October 2017 were used, for a total of 190 images at a rate of 22 to 24 images per year. Based on previous studies done on Lake Winnipeg, lake pixels with chlorophyll-a concentrations in excess of 10 mg/m^3^ were assumed to signal the presence of significant phytoplankton biomass^22–24^. In each of the lake basins, bloom conditions were inferred when chlorophyll-a concentrations in excess of 10 mg/m^3^ were detected for 33% of the available lake pixels. The 33% criterion was used here to align with a recent study based on MERIS satellite images and which showed that over the 2002–2009 period (i.e., the period preceding our analysis), phytoplankton blooms in Lake Winnipeg covered, on average, 7832 km^2^, which corresponds to 32.9% of lake pixels^41^. It should be noted that in our study, due to the 4-km resolution of the MODIS images, good spatial coverage was available for the north basin of Lake Winnipeg, while only partial data was obtained for the south basin and no data for the narrows connecting the two basins. Consequently, while assessments of bloom occurrence were done for both basins (Fig. 2a,b), only the assessments based on the larger north basin with good data coverage were used to identify moderate-bloom years and major-bloom years.

Digital elevation data and land use and land cover datasets for the Lake Winnipeg Watershed were downloaded from the Government of Canada open data website (https://open.canada.ca/en). For the characterization of surface and near-surface hydrologic connectivity in the Lake Winnipeg Watershed, weekly percent saturated surface soil moisture maps derived from SMOS (Soil Moisture and Ocean Salinity) data were obtained. Level 2 gridded SMOS products have an inter-pixel distance of approximately 15 km. Similar to the MODIS/Terra data mentioned above, SMOS data were used here because of their global coverage, multi-year availability (2009–2017) and open access. SMOS data were downloaded from the Government of Canada open data website (https://open.canada.ca/data/en/dataset/c0ea8c27-e62e-45bc-b64c-d475650d84a2) and fall under an Open Government License (https://open.canada.ca/en/open-government-licence-canada). Due to the very large area of the Lake Winnipeg Watershed, however, some of the weekly SMOS maps were discarded when soil moisture values were missing for a large number of pixels. A total of 165 SMOS maps covering the whole spatial extent of the Lake Winnipeg Watershed were selected for the period May 2010 to October 2017, at a rate of 19 to 25 per year. For each week under consideration, watershed pixels were labelled as hydrologically inactive or hydrologically active by transforming the continuous soil moisture map into an indicator map. The latter is a binary map where 0s signal pixels for which soil moisture is below a threshold value, while 1s flag pixels for which soil moisture is above a threshold value. Previous studies that relied on soil moisture indicator maps used a uniform threshold value across whole watersheds^20,21,42^. However, in order to account for the spatial variability in soil properties present across the Lake Winnipeg Watershed, a pixel-specific threshold value, equal to the topsoil field capacity, was used in our study. Field capacity is a critical soil hydraulic property that refers to the soil moisture value above which water is no longer held in the soil and available to flow, and it depends on soil properties such as texture (i.e., proportion of sand, silt, clay) and organic matter content. Across the Lake Winnipeg Watershed, spatially detailed information about soil texture and organic matter information was extracted from the peer-reviewed, open-access Unified North American Soil Map^43^. Commonly used pedotransfer functions^44^ were then used to predict field capacity from topsoil properties. Pixels with percent soil moisture values above their field capacity threshold were deemed hydrologically active (i.e., runoff-producing), while others were deemed inactive. Since SMOS data only capture near-surface (<5 cm) soil moisture, hydrologic activity, when detected, was attributed to surface or shallow subsurface runoff activation. The relatively coarse-resolution of the satellite-derived soil moisture maps (i.e., 15 km) also means that hydrologic activity was detected only when it occurred over relatively large swaths of land (i.e., covering 125 km^2^ or more), as opposed to single farm fields or complexes of small prairie pothole wetlands, for instance. This drawback may explain why very few hydrologically active and hydrologically connected areas were identified in Western Manitoba and Eastern Saskatchewan, two provinces dominated by small topographic depressions and depressional wetlands. Hydrologic connectivity was inferred when hydrologically active areas were spatially contiguous to (i.e., adjacent to or “touching”) a major river in the Lake Winnipeg Watershed. The phrase “major river” refers to waterways, mostly natural, that are mapped at the provincial and federal level, typically at the 3^rd^ order (Strahler) and higher. Smaller natural waterways, as well as artificial (i.e., man-made) drainage channels, were excluded, not for lack of importance but rather because of inconsistent mapping across jurisdictions.

## Supplementary information

Supplementary video

## Data Availability

All data analyzed in the current study are available through online, open data repositories, as specified in the Methods section.
